# The wheat *Phs-A1* pre-harvest sprouting resistance locus delays the rate of seed dormancy loss and maps 0.3 cM distal to the *PM19* genes in UK germplasm

**DOI:** 10.1093/jxb/erw194

**Published:** 2016-05-23

**Authors:** Oluwaseyi Shorinola, Nicholas Bird, James Simmonds, Simon Berry, Tina Henriksson, Peter Jack, Peter Werner, Tanja Gerjets, Duncan Scholefield, Barbara Balcárková, Miroslav Valárik, M. J. Holdsworth, John Flintham, Cristobal Uauy

**Affiliations:** ^1^John Innes Centre, Norwich Research Park, NR4 7UH, UK; ^2^KWS UK Ltd, Hertfordshire, SG8 7RE, UK; ^3^Limagrain UK Ltd, Woolpit Business Park, IP30 9UP, UK; ^4^Lantmannen, SE-268 81, Svalov, Sweden; ^5^RAGT Seeds, Essex, CB10 1TA, UK; ^6^Division of Plant and Crop Sciences, School of Biosciences, University of Nottingham, LE12 5RD, UK; ^7^Institute of Experimental Botany, Centre of the Region Haná for Biotechnological and Agricultural Research, Šlechtitelů 31, 78371 Olomouc, Czech Republic

**Keywords:** After-ripening, dormancy, PM19, pre-harvest sprouting, seed, synteny, *Triticum*, *aestivum*.

## Abstract

The precocious germination of cereal grains before harvest, also known as pre-harvest sprouting, is an important source of yield and quality loss in cereal production. Pre-harvest sprouting is a complex grain defect and is becoming an increasing challenge due to changing climate patterns. Resistance to sprouting is multi-genic, although a significant proportion of the sprouting variation in modern wheat cultivars is controlled by a few major quantitative trait loci, including *Phs-A1* in chromosome arm 4AL. Despite its importance, little is known about the physiological basis and the gene(s) underlying this important locus. In this study, we characterized *Phs-A1* and show that it confers resistance to sprouting damage by affecting the rate of dormancy loss during dry seed after-ripening. We show *Phs-A1* to be effective even when seeds develop at low temperature (13 °C). Comparative analysis of syntenic *Phs-A1* intervals in wheat and *Brachypodium* uncovered ten orthologous genes, including the *Plasma Membrane 19* genes *(PM19-A1* and *PM19-A2*) previously proposed as the main candidates for this locus. However, high-resolution fine-mapping in two bi-parental UK mapping populations delimited *Phs-A1* to an interval 0.3 cM distal to the *PM19* genes. This study suggests the possibility that more than one causal gene underlies this major pre-harvest sprouting locus. The information and resources reported in this study will help test this hypothesis across a wider set of germplasm and will be of importance for breeding more sprouting resilient wheat varieties.

## Highlight

*Phs-A1* confers resistance to sprouting in wheat by delaying the rate of seed dormancy loss and is distinct from the previously proposed *PM19* candidate genes.

## Introduction

Pre- and post-harvest crop losses caused by biotic or abiotic stress factors are major drawbacks to attaining global food security. In addition to their detrimental effects on crop yield, their effects on quality are equally damaging. Pre-harvest sprouting (PHS) represents one such source of both yield and quality loss in global wheat production. PHS is characterized by the precocious germination of grains before harvest with consequent reductions in seed viability and end-use value, particularly for bread-making purposes. PHS is strongly influenced by the environment and is especially prevalent in wheat-growing regions with high rainfall during the period of grain maturation and ripening (Mares and Mrva, 2014). In addition, adverse environmental conditions like heat stress or water deficit during grain development have been generally associated with higher levels of seed germination upon grain maturation and this predisposes plants to incidences of PHS (Rodriguez *et al*, 2011). Given the current climate change projections of increased temperature and precipitation in parts of the world (Walther *et al.*, 2002; Trenberth, 2011), the incidence of PHS is expected to increase and become a greater challenge in wheat production areas.

Breeding for PHS resistance is an effective and environmentally sustainable strategy to address this problem. This can be achieved by breeding for seed dormancy, which is the most dominant component of PHS resistance. In general, plants with higher seed dormancy and responsiveness to abscisic acid (ABA) – a key hormone regulating seed dormancy – display higher sprouting resistance (Walker-Simmons, 1987; Gerjets *et al.*, 2010). On the other hand, prolonged seed dormancy is not desirable as it delays growing cycles and results in non-uniform germination upon sowing. The desired breeding objective is therefore an intermediate level of seed dormancy that confers resistance to sprouting during seed development and maturation, but declines shortly thereafter. However, breeding for PHS resistance is made difficult by the highly quantitative nature of this trait: numerous quantitative trait loci (QTL) accounting for varying degrees of resistance have been identified on all 21 wheat chromosomes (reviewed in Flintham *et al.*, 2002; Gao *et al.*, 2013b; Mares and Mrva, 2014), highlighting the complexity of its genetic control.

Despite this, a few QTL have been consistently identified across diverse germplasm which account for a large proportion of the sprouting variation observed in modern wheat cultivars. These loci include the *Red grain* colour genes (*R-1*) on the long arm of group 3 chromosomes, which encode a Myb transcription factor (*TaMyb10*) that regulates flavonoid biosynthesis (Himi and Noda 2005; Himi *et al.* 2011). Although not well understood, *TaMyb10* also has pleiotropic effects on seed dormancy, with red-grained wheat being generally, but not always, more dormant than white-grained wheat (Mares *et al.* 2005; Kottearachchi *et al.*, 2006; Mares and Mrva 2014). Besides the *R-1* loci, two major QTL on chromosome arms 3AS and 4AL account for a considerable proportion of the variation in PHS resistance when independent of grain colour. The 3AS QTL was recently cloned and shown to encode for Mother of Flowering Time (TaMFT: Nakamura *et al.*, 2011; Lei *et al.*, 2013; Liu *et al.*, 2013b), which promotes seed dormancy in wheat embryos. *TaMFT* is upregulated when seeds develop at temperatures below 13 °C, suggesting its involvement in the integration of environmental inputs into the regulation of seed dormancy (Nakamura *et al.*, 2011).

The 4AL QTL for PHS resistance was first reported by Flintham (2000) and was originally named *Phs*, although here we refer to it as *Phs-A1* in agreement with the current wheat gene nomenclature. This locus was subsequently identified in diverse germplasm (Imtiaz *et al.*, 2008; Ogbonnaya *et al.*, 2008; Torada *et al.*, 2008). In these studies the QTL effect was rarely characterized, thus limiting the understanding of its physiological basis. Also, the gene(s) underlying *Phs-A1* have recently been investigated. A number of candidate genes including those encoding for Aquaporin (Lohwasser *et al.*, 2013) and GA20-Oxidase (Tyagi and Gupta, 2012; Cabral *et al.*, 2014) have been suggested based on homology to model species. Recently a transcriptomics study of wheat seed dormancy by Barrero *et al.* (2015) identified two tandem genes, *PM19-A1* and *PM19-A2*, encoding ABA-inducible Plasma Membrane 19 proteins, as the main candidates for *Phs-A1*. Furthermore, the *PM19* genes were shown to be positive regulators of wheat seed dormancy as RNA interference (RNAi) lines with reduced expression of both genes show reduced level of seed dormancy (Barrero *et al.*, 2015).

In the present study we identified, validated and characterized the effect of the 4AL *Phs-A1* QTL. We show that this QTL exerts its effect by regulating the duration of seed dormancy loss after seed maturation. Furthermore, through multiple fine-mapping experiments in independent bi-parental mapping populations, we narrowed down the effective resistance locus to an interval less than 0.5 cM which, surprisingly, excludes the *PM19* candidate genes reported by Barrero *et al.* (2015). We discuss the conflicting nature of these results and the implication of our finding on the effort to breed for more PHS resilient wheat varieties.

## Materials and methods

### Plant materials

The identification, characterization and high-resolution fine-mapping of *Phs-A1* were done in two experimental populations made from the Option × Claire and Alchemy × Robigus crosses. These four cultivars are UK winter bread-wheat varieties with Alchemy and Option being PHS resistant while Claire and Robigus are PHS susceptible. The 4AL *Phs-A1* QTL was originally detected in the doubled haploid (DH) populations made from these crosses using the wheat × maize technique from F_1_ plants (Laurie and Bennett, 1988). Forty-eight and 122 DH individuals were analysed in the Alchemy × Robigus and the Option × Claire population, respectively. Selected DH lines from these populations were subsequently used to develop the mapping population used in this study as detailed below.

#### Alchemy × Robigus fine-mapping population

For subsequent characterization and fine mapping of *Phs-A1* in the Alchemy × Robigus population, we developed near isogenic lines (NILs) and recombinant inbred lines (RILs). To accomplish this, five SSR markers including *barc170, wmc420, wmc707*, *wmc760* and *wmc313 were* used to select DH lines homozygous for Alchemy in different, but overlapping, intervals across the 4AL chromosome arm. These were independently backcrossed to the recurrent parent Robigus and advanced to the BC_3_ generation by crossing heterozygous plants selected at each generation. Following self-pollination of selected BC_3_F_1_ lines, NILs homozygous for the Alchemy introgression found in the original DH lines were selected using the SSR markers flanking the introgressions. For the development of RILs used for high resolution fine-mapping, BC_3_F_2_ lines heterozygous for *Phs-A1* interval (*barc170-wmc420*) were self-pollinated and BC_3_F_3_ lines with recombination events between the critical *Phs-A1* interval were selected. These were advanced to the BC_3_F_4_ generation by self-pollination to obtain homozygous RILs.

#### Option × Claire fine-mapping population

We also developed F_4_ RILs from the Option × Claire cross. This was accomplished by crossing a DH line (OC69) homozygous for Option across the QTL interval with Claire. Following self-fertilization of F_1_ progeny, 2400 F_2_ plants were screened and 85 F_2_ recombinant lines with recombination events between markers *barc170, wms894 and xhbe03* were recovered. Thirty of these lines were randomly selected, self-fertilized and lines with homozygous recombinant haplotype were extracted from the F_3_ population. In addition, lines with Claire or Option non-recombinant haplotype were also selected as controls. However, only 27 of these were initially phenotyped and advanced to the F_4_ generation for further phenotyping.

### Growth conditions

Three germination index (GI) experiments and five sprouting experiments were conducted in the Alchemy × Robigus and the Option × Claire populations. All the GI experiments were conducted in the Alchemy × Robigus population, whereas sprouting experiments were conducted in both the Alchemy × Robigus (sprouting experiment-1 and 5) and Option × Claire populations (sprouting experiment-2, -3 and -4). In GI experiment-1 and sprouting experiment-1, -3 and -5, plants were grown in the glasshouse under long day conditions with 16h light (300 mmol) at 18 °C, 8h darkness at 15 °C and at relative humidity of 70%. In GI experiment-2 and sprouting experiment-4, plants were grown in controlled environment room (CER) under long day conditions with 16h light (250–400 mmol) at 20 °C, 8h darkness at 15 °C and at 70% relative humidity. GI experiment-3 was designed to test if *Phs-A1* is still effective when grains are developed at low temperature. In this experiment, plants were transferred 1–7 d after anthesis into a CER and maintained at constant day and night temperature of 13 °C. Plant materials for sprouting experiment-2 were grown in the field at Thriplow, UK (52.1000° N, 0.1000° E) as single rows in 1 m^2^ plots using in a randomized complete block design with two replications per line.

### SNP and SSR genotyping

Five SSR markers including *wmc420, barc170, wmc707, wmc760* and *wmc313* were used for the development of Alchemy × Robigus NILs. For the fine-mapping of *Phs-A1, SSR wms894* and *xhbe03* were used to genotype Option × Claire RILs while only x*hbe03* was used for Alchemy × Robigus RILs as *wms894* is not polymorphic in this cross. The primer sequences of SSR markers were obtained from the GrainGenes database (http://wheat.pw.usda.gov/GG3, last accessed 4 February 2016), except for *wms894* which was obtained from RAGT Seed, UK. These were labelled with the FAM, VIC, NED or PET fluorescent dye (Applied Biosystems) for the multiplexing of assays. PCR were performed with the Qiagen Hotstart Master Mix (Qiagen, Cat No: 203443) and in volume of 6.25 µl containing 3.125 µl of Hotstart mix, 0.625 µl of primer mix and 2.5 µl of DNA. Thermal cycling conditions was as follow: Hotstart at 95 °C for 15min, 35 cycles of 95 °C for 1min; 50–60 °C (depending on annealing temperature of primers) for 1min, 72 °C for 1min and a final extension step of 72 °C for 10min. PCR amplicon were afterwards run on an Applied Biosystems 3730 DNA Analyzer using GeneScan 500 LIZ (Thermo Fisher Scientific; Cat. No:4322682) as size standard. Genotype data were analysed on the GeneScan® Analysis Software (Applied Biosystems). SSR markers *barc170, wmc707, wmc760* and *wmc313* gave Alchemy/Robigus band sizes of 170/180bp, 165/190bp, 110/100bp and 185320/340325bp, respectively, while *wms894* and *xhbe03 gave* Option/Claire band sizes of 160/125bp and 140/138bp, respectively.

For the development of SNP markers, sequences of wheat genes orthologous to *Brachypodium* genes in the syntenic *Phs-A1* interval were amplified and sequenced to identify SNPs between parental lines. Kompetitive Allelic Specific PCR assays (KASP: Smith and Maughan, 2015) were developed for each SNP. Assays were performed in 384 well plate format in a 5.07 µl volume containing 2.5 µl of DNA, 2.5 µl of KASP master mix (LGC, UK) and 0.07 µl of primer mix. PCR was performed on an Eppendorf Mastercycler pro 384 using the following protocol: Hotstart at 95 °C for 5min, ten touchdown cycles (95 °C for 20s; touchdown 65 °C, −1 °C per cycle, 25s) followed by 30–40 cycles of amplification (95 °C for 10s; 57 °C for 1min). No extension step is necessary as KASP amplicons are smaller than 100bp. Plates were read using the Tecan SAFIRE Fluorescent Scanner and genotype data was viewed graphically with the KlusterCaller™ software (LGC, UK).

### Germination index (GI) assays

At four stages of grain maturation and after ripening including physiological maturity, harvest maturity (7 d after physiological maturity), as well as 14 and 28 d after harvest maturity, ears were harvested and gently threshed to obtain grains from the central portion of the ear. For plants grown under constant 13 °C in GI experiment-3, harvest maturity was reached 12 d after physiological maturity. Twenty grains were placed with the crease facing down in 90mm petri dishes containing two layers of Sartorius filter paper and were incubated in 5ml of sterile water for 7 d. After each day of incubation, germinated seeds (with ruptured seed coat) were counted and removed from the plate. The number of germinated seeds per day was used to calculate a weighted GI score using the formula described by Walker-Simmons (1987) with a slight modification: GI=(7×*n*1)+(6×*n*2)+(5×*n*3)+(4×*n*4)+(3×*n*5)+(2×*n*6)+(1×*n*7)/7×(N−M). Where *n1, n2, … n7* are the number of germinated grains on the first, second, and nth days until the 7th day, respectively; N is the total number of grains per plate and M is the number of mouldy grains after the 7 d of incubation. GI test were conducted on ears from six independent plants per NIL group in GI experiment-1 and three to four independent plants per line in GI experiment-2 and -3. The average GI scores are presented.

### Sprouting test

Plants were synchronized by days to flowering and maturity and ears were harvested at similar times post-anthesis and allowed to after-ripen at room temperature (20 °C) until GI differences were observed between the parental varieties. After-ripened ears (two to three per plant) were arranged standing upright on wire racks loaded on a revolving wheel inside a sprouting chamber. The ears were then misted for 5–7 d under 100% humidity. Misted ears were dried and ears from the same plant were gently threshed together to collect grains, which were examined for the symptoms of sprouting damage (breakage of the seed coat near the embryo). This was used to calculate the percentage of sprouting in each plant. The number of independent plants phenotyped per line ranged from 12 plants per NIL in sprouting experiment-1; two plants per RIL in sprouting experiment-2, 2–6 plants per RIL in sprouting experiment-3; 4–24 independent plants per RIL in sprouting experiment-4 and three plants per RIL in sprouting experiment-5. The total number of seeds across the experiments ranged from 50 to 200 seeds per genotype. The NILs and RILs were grouped according to their haplotype and the number of lines in each group are indicated in the figure legends. The average sprouting percentages of haplotype groups are presented in the main figures while the average sprouting percentages of individual RILs in experiments-3 to -5 are presented in the supplementary data.

### Statistical analyses

Statistical significance was calculated using either one-way or two-way analyses of variance (ANOVA). Tukey’s Honestly Significant Difference (HSD) tests and Dunnett’s tests (using parental varieties as controls) were performed for multiple comparisons between NIL, RILs and parents. Data that did not meet the ANOVA assumption of homogeneity of variance were arcsin transformed and confirmed to meet the assumptions before being used for the ANOVA analysis. Statistical analyses were performed in Genstat (version15.2.0.8821) and Minitab (Version, 17.2.1).

## Results

### Validation of *Phs-A1* in UK bi-parental populations

**Fig. 1.**
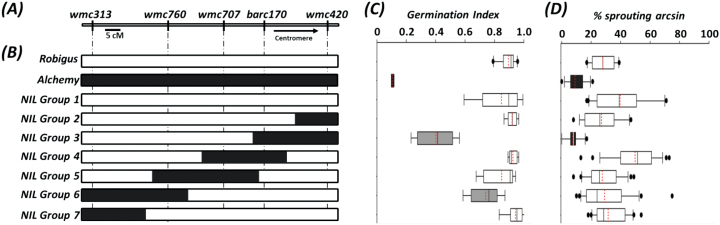
Validation of *Phs-A1* in Alchemy × Robigus NILs. (A) Genetic map of SSR markers across the 4AL chromosome arm used to develop the NILs. (B) Graphical genotypes of Alchemy × Robigus NILs. The NILs are grouped based on their recombination haplotype across the marker intervals, with each group comprising two independent NILs. The black filled portion in the graphical genotype represents the Alchemy alleles, whereas the white sections represent the Robigus alleles. (C) Mean germination index of each NIL group in GI experiment-1. (D) Sprouting phenotype of each NIL group in sprouting experiment-1. The left and right boundaries of the boxplot indicate the 25th and 75th percentile, respectively, while the error bars (whiskers) on either side of the boxplot indicate the 10th and 90th percentiles. The solid line within the boxplot marks the median (50th percentile) while the red line within the box marks the mean.

The 4AL QTL effect is known to segregate in UK wheat varieties (Flintham *et al.*, 2011) and has been identified in DH populations derived from crosses between Alchemy × Robigus and Option × Claire (Alchemy and Option providing the resistance allele). The QTL is flanked by markers *barc170* and *wmc491* in the Alchemy × Robigus population (Supplementary Fig. S1 at *JXB* online) and as such collocated with the major *Phs-A1* QTL identified across multiple studies (Flintham *et al.*, 2002; Imtiaz *et al.*, 2008; Torada *et al.*, 2008; Barrero *et al.*, 2015). To independently validate the effect of *Phs-A1*, we developed NILs from the Alchemy × Robigus cross through marker-assisted backcrossing (details in Methods). Five markers distributed across the 4AL chromosome arm were used for NIL development, including *barc170* and *wmc420* (1 cM proximal to the *wmc491* flanking marker) as well as *wmc707*, *wmc760* and *wmc313* which are distal to *barc170* (Fig. 1A). Seven overlapping recombination haplotypes (designated as NIL Groups 1–7) were developed (Fig. 1B) with only Group 3 NILs containing the Alchemy resistant haplotype across the complete QTL interval (*barc170*–*wmc420*).

We assessed the seed dormancy and PHS resistance phenotype of these NILs through a GI test on threshed seeds (GI experiment-1) and an artificial sprouting test on whole spikes (sprouting experiment-1). In the GI test, highly significant differences were observed between the Robigus and Alchemy parental controls (*P*<0.001; Fig. 1C). NILs were classified as either resistant or susceptible based on a Dunnett’s test to the parental controls. NILs with higher or non-significant GI differences than Robigus were classified as susceptible, while NILs with lower or non-significant GI differences than Alchemy were classified as resistant (Supplementary Table S1). NIL Groups 1, 5 and 7 with the Robigus haplotype across the QTL interval, and NIL Groups 2 and 4 with recombinant haplotypes within the QTL interval, all showed the susceptible GI phenotype (Fig. 1C). Group 3 NILs showed significantly lower GI than Robigus (*P*<0.001) but also significantly higher GI than Alchemy (*P*<0.001). Likewise, Group 6 NILs also showed significant differences from both parents but the GI was only slightly lower than the susceptible Robigus parent.

In the sprouting test, all the NIL groups (except Group 3) were significantly different than Alchemy but not Robigus and were therefore classified as being susceptible to sprouting (sprouting experiment-1; Fig. 1D). Group 3 NILs showed comparable sprouting levels to the resistant variety Alchemy, and were significantly different from Robigus (*P*<0.001), consistent with the GI results. Taken together, the GI and sprouting results validate the resistance effect of *Phs-A1* in NILs with the Alchemy haplotype across the complete *barc170*-*wmc420* interval. NILs from Group 2 and 4, which have the Alchemy allele at either one or other – but not both – flanking markers, were susceptible suggesting that the *Phs-A1* resistance locus is delimited by, but not linked to, these markers.

### *Phs-A1* reduces the rate of dormancy loss during dry seed after-ripening

The validation experiments suggest that *Phs-A1* confers PHS resistance by affecting seed dormancy since the effect was not only observed in whole spikes (sprouting test), but was also evident in the reduced germination potential of threshed seeds (GI test). To further understand this resistance mechanism, we examined the rate of dormancy loss in one of the resistant and susceptible NIL groups used in the previous experiment through the GI test (GI experiment-2). This was done across different stages of seed maturation and after-ripening including physiological maturity (PM, ~40% seed moisture content), harvest maturity (HM, ~20% seed moisture content) and two post-harvest time points (14 and 28 d post-harvest, DPH). The NILs used for the experiment (NIL Group 2 and 3) were genetically isogenic and only differed in the extent of the Alchemy introgression between *barc170* and *wmc420* (Fig. 1A). This allows for a precise characterization of this region without the confounding effect of other background loci.

**Fig. 2.**
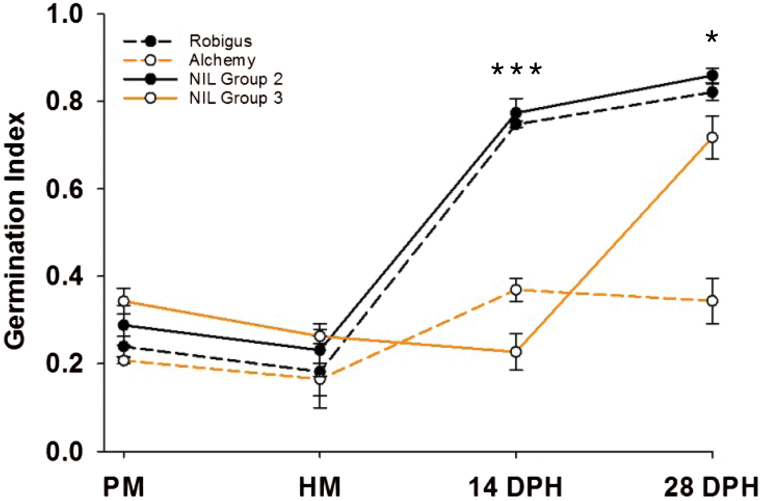
*Phs-A1* delays the rate of seed dormancy loss during after-ripening. The germination index of seeds harvested from Robigus, Alchemy and NILs with either the recombinant haplotype (NIL Group 2) or Alchemy haplotype (NIL Group 3) between *barc170* and *wmc420* in GI experiment-2. Seeds were tested at physiological maturity (PM), harvest maturity (HM; 7 d after PM), 14 and 28 d post-harvest (DPH) and germinated at 16 °C. Error bars represent standard error of the means (SEM) of three biological replications for each time point. Significant differences between NILs at *P*<0.05 (*) and *P*<0.001 (***) are indicated.

At PM and HM, all lines showed similarly low GI with no difference between the contrasting alleles (*P*>0.51; Fig. 2). However, at 14 DPH there was a significant GI difference between the contrasting NILs and parents, with Robigus and the susceptible Group 2 NIL showing increased germination potential compared to Alchemy and the resistant Group 3 NIL (*P*<0.001). This difference in germination potential was maintained at 28 DPH, although the differences were reduced and less significant than at 14 DPH (*P*<0.05).

The previously cloned seed-coat-independent PHS QTL regulated by *TaMFT* is effective when seeds develop under low temperature (Nakamura *et al.*, 2011). We therefore examined if *Phs-A1* was effective at low temperature by measuring the rate of seed germination in NILs grown at 13 °C post-anthesis (GI experiment-3). While the NILs displayed an overall higher depth of seed dormancy when plants were grown at 13 °C post anthesis, the same pattern of GI differences at the post-harvest time-points (but not at PM and HM) was observed (Supplementary Fig. S2). Together, these experiments suggest that *Phs-A1* delays the rate of seed dormancy loss during after-ripening.

### *Phs-A1* maps to a 0.5 cM interval between wms894 and xhbe03

**Fig. 3.**
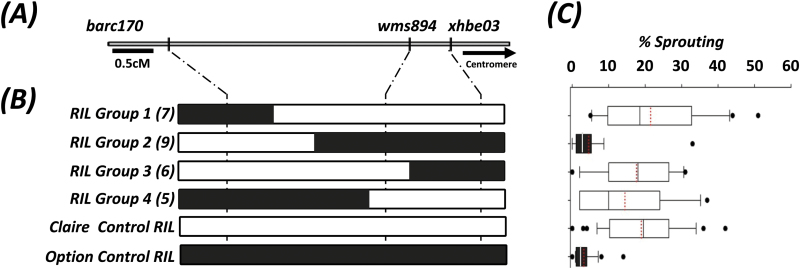
Interval mapping of *Phs-A1* in the Option × Claire RIL population. (A) Genetic map of the SSR markers flanking *Phs-A1*. (B) Graphical genotypes of RILs and controls are presented with the Option and Claire alleles represented in black and white, respectively. The RILs are grouped according to their fixed genotype across the *Phs-A1* interval and the number of lines in each RIL group is indicated in parenthesis. (C) Sprouting phenotype of RIL groups and controls in sprouting experiment-3. The left and right boundaries of the boxplot indicate the 25th and 75th percentile, respectively, while the error bars (whiskers) on either side of the boxplot indicate the 10th and 90th percentiles. The solid line within the boxplot marks the median (50th percentile) while the red line within the box marks the mean. Boxes with the same colour are similar to each other based on pairwise comparisons.

As previously stated, *Phs-A1* is flanked by *barc170* and *wmc420* in the bi-parental populations used in this study. Torada *et al.* (2008) mapped *Phs-A1* to a 2.6 cM interval between *barc170* and *xhbe03*. We therefore used *barc170*, *xhbe03*, and another marker – *wms894* – in the same physical bin (4AL_13-0.59–0.66), to characterize Option × Claire F_4_ RILs (Fig. 3A, B). We selected 27 homozygous recombinants across the interval, grouped these according to their haplotypes (Fig. 3B), and assessed the sprouting phenotype using the artificial sprouting test (sprouting experiment-2). Two significantly different sets were identified in this experiment: one was made up of RIL Group 2 and Option Control RILs with between 3 and 5% sprouting, whereas the second set contained RIL groups 1, 3, 4 and the Claire Control RILs with average sprouting between 15 and 22% (Fig. 3C). RIL Group 2 and the Option Control RILs were similar to the resistant Option parent and had the Option haplotype between *wms894* and *xhbe03*. RIL Groups 1, 3, 4 and the Claire Control RILs were similar to the susceptible Claire parent and carried a homozygous Claire or recombinant haplotype across the *wms894–xhbe03* interval. This suggests that the *Phs-A1* resistance is only observed when RILs have the Option haplotype across the 0.5 cM *wms894-xhbe03* interval.

### Synteny reveals the putative gene content of the *Phs-A1* locus

Given the small genetic interval to which *Phs-A1* mapped, we evaluated the gene content across this locus. We first identified genes containing the flanking markers (*xhbe03* and *wms894*) in wheat: *xhbe03* is designed from the 3ʹ UTR sequence of *PM19-A2* (*Traes_4AL _F99FCB25F*) while the sequence of *wms894* is located in the promoter region of an *OTU Cysteine Protease* gene (*Traes_4AL _F00707FAF*). We next examined the collinear region in *Brachypodium*: reciprocal BLASTs against the *Brachypodium* genome identified *Bradi1g00600* and *Bradi1g00720* as orthologues of *PM19-A2* and *OTU Cysteine Protease,* respectively. This defined the collinear *Phs-A1* interval in *Brachypodium* to a 75kb region which contains 11 genes (*Bradi1g00607* to *Bradi1g00710*).

**Fig. 4.**
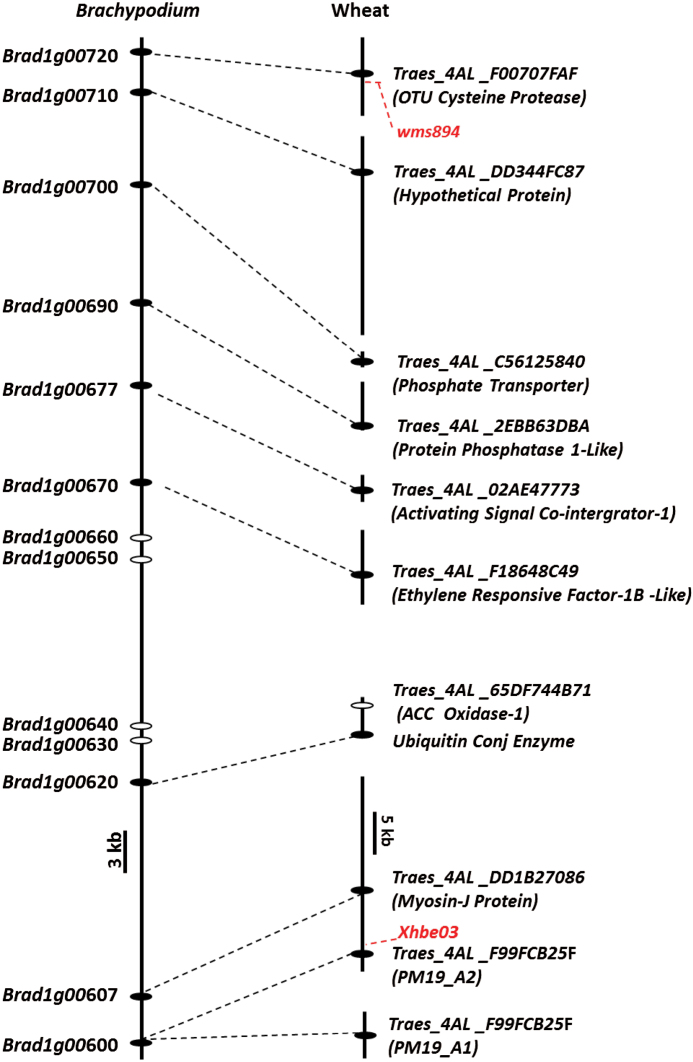
Synteny reveals the putative gene content of the *Phs-A1* locus. Sequences of genes containing the *Phs-A1* flanking markers (*wms894* and *xhbe03*; in red) were used to obtain genes in the orthologous *Brachypodium* interval (*Brad1g00600*–*Brad1g0072*0). Collinear genes are represented by black ovals while non-collinear genes are represented by the white ovals. Orthologous wheat contigs (black lines) and gene models are connected to their corresponding *Brachypodium* genes. All the wheat genes were genetically mapped within or linked to the *wms894–xhbe03* interval except for *Traes_4AL _C56125840.* Wheat orthologue could not be found for *Brad1g00630–Brad1g00660.*

The *Brachypodium* genes were used to search the wheat chromosome arm assemblies of the hexaploid wheat cultivar, Chinese Spring (IWGSC, 2014). Orthologous contigs and gene models to *Bradi1g00600–Bradi1g00620* and *Bradi1g00670–Bradi1g00720* were identified on chromosome arm 4AL. These included *PM19-A2* and its paralogue *PM19-A1,* as well as genes encoding for Myosin J protein, Ubiquitin conjugating enzyme, Ethylene Responsive Factor-1B-Like (ERF-1B-Like), Activating Signal Co-integrator-1 (ASC1), Protein Phosphatase 1-Like (PP1-Like), a phosphate transporter, a hypothetical protein and the OTU Cysteine Protease (Fig. 4, Supplementary Table S2). No wheat orthologues were identified for *Bradi1g00630* to *Bradi1g00660*. Within the wheat IWGSC contigs, a non-collinear gene encoding for an Aminocyclopropane Carboxylate Oxidase 1-Like protein (ACC Oxidase-1; *Traes_4AL_65DF744B71*) was also identified. All these genes/contigs were mapped within or linked to the critical *wms894–xhbe03* interval using SNP-based KASP assays (Supplementary Table S3), except for *Traes_4AL_C56125840* which we did not map due to the lack of a genetic marker. This confirmed the collinear gene order between wheat and *Brachypodium* and suggests possible candidate genes for *Phs-A1*.

### *Phs-A1* maps distal to the PM19 genes in two UK fine-mapping populations

**Fig. 5.**
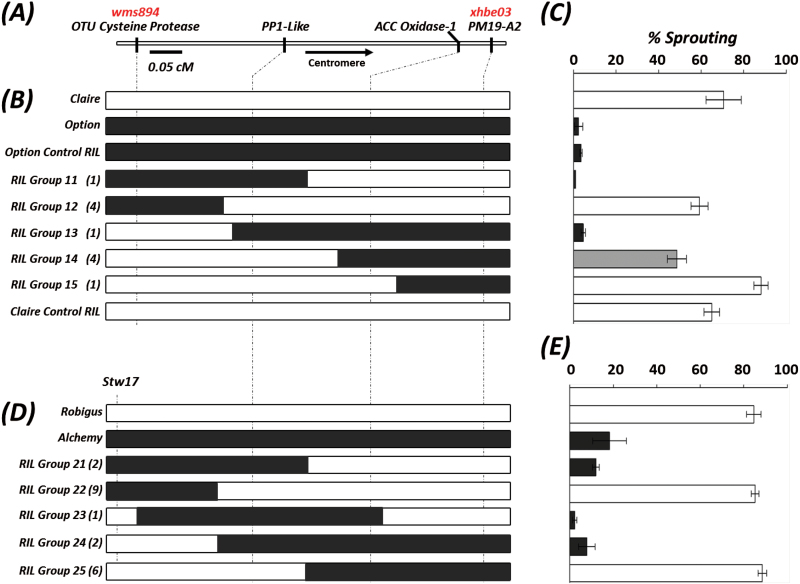
High-resolution fine-mapping of *Phs-A1* in Option × Claire and Alchemy × Robigus RIL populations. (A) Linkage map of SNP (black) and SSR (red) markers across the *Phs-A1* interval. The graphical genotype of Option × Claire RILs (B) and Alchemy × Robigus RILs (D) are aligned against their sprouting phenotype (C and E, respectively). RILs are grouped based on their recombination haplotype across the marker interval and the number of lines in each group is indicated in parentheses. Resistant parent alleles (Option and Alchemy) are represented in black, whereas the susceptible parent alleles (Claire and Robigus) are shown in white. Marker *stw17* (2 cM distal to *wms894*) was used in the Robigus × Alchemy population as *wms894* and OTU Cysteine Protease are monomorphic. The sprouting phenotype of each RIL group is designated as susceptible (white), moderate (grey) or resistant (black) based on statistical comparison with the parental controls. Error bars represents SEM.

Barrero *et al.* (2015) identified *PM19-A1* and *PM19-A2* as the main candidates for a seed dormancy QTL on wheat 4AL chromosome arm in a multi-parental mapping population. To determine if these genes determined the allelic variation observed in the UK populations, we further fine-mapped *Phs-A1* in the Option × Claire F_4_ RILs with homozygous recombinant and non-recombinant haplotypes in the *Phs-A1* interval. We first defined the linkage between the gene-based KASP assays previously used to map the syntenic genes (Fig. 5A). The two *PM19* genes were completely linked and so too were the *PP1-like*, *ERF-1B-like* and *ASC1* genes. There were however recombination events between *PP1-Like/ ERF-1B-Like/ ASC1* and *OTU Cysteine Protease* and between *ACC Oxidase-1* and *PM19-A2/PM19-A1*. Given the genetic linkage between some of these markers, four SNP markers including *OTU Cysteine Protease*, *PP1-Like, ACC Oxidase-1* and *PM19-A2* (Supplementary Table S3) were used to define five distinct recombinant haplotypes (RIL Group 11–15; Fig. 5B).

A subset of lines from each RIL group, in addition to the parental cultivars and non-recombinant Claire and Option control RILs were phenotyped using the artificial sprouting test (sprouting experiment-3; Fig. 5B). Variation in sprouting percentage was observed defining a bimodal distribution (Supplementary Fig. S3). To unequivocally assign sprouting phenotypes to these lines, the mean sprouting percentages of each RIL group (Fig. 5C) as well as the individual sprouting percentages of each RIL (Supplementary Table S4) were compared against those of Claire and Option using the Dunnett’s test. This showed that the sprouting phenotype is completely associated to the *PP1-Like*/*ERF-1B-Like*/*ASC1* linkage in all the lines tested. Five independent recombination events (Group 12 and 13) map *Phs-A1* proximal to the *OTU Cysteine Protease* (*wms894*) marker. Similarly, the six lines from RIL Groups 11, 14 and 15 map *Phs-A1* distal to both the *ACC Oxidase-1* and the *PM19* genes. This was unexpected given the reported association of the *PM19* genes with sprouting resistance. We confirmed this result in an independent sprouting experiment (sprouting experiment-4; Supplementary Table S5) using the critical Group 12–14 RILs. Although a higher level of sprouting was observed in this experiment, *Phs-A1* still conferred a moderate level of resistance, which was associated with the *PP1-Like*/*ERF-1B-Like*/*ASC1* linkage.

We also independently fined-mapped *Phs-A1* in an Alchemy × Robigus RIL population, which contained similar recombination haplotypes (RIL Groups 21–25; Fig. 5D) as in the Option × Claire population. However, marker *stw17* was used in place of the *OTU Cysteine Protease* marker as this was not polymorphic in the Alchemy × Robigus cross. We assessed the sprouting phenotype of these lines using the artificial sprouting test (sprouting experiment-5, Supplementary Table S6). Similar to the previous results, the mean sprouting percentages of each RIL group (Fig. 5E) confirmed the complete linkage of *Phs-A1* to *PP1-Like*, *ERF-1B-Like* and *ASC1* genes. Eleven independent recombination events in RIL Group 22 and 24 map *Phs-A1* proximal to *stw17*, whereas nine independent RILs map *Phs-A1* distal to the *ACC Oxidase-1* and *PM19* genes (RIL Groups 21, 23 and 25). This provides strong genetic evidence that in the two UK mapping populations *Phs-A1* maps distal to the *PM19* genes.

## Discussion

### *Phs-A1* confers resistance to sprouting in wheat

In this study we characterized and fine-mapped *Phs-A1*, a major PHS resistance and seed dormancy QTL in wheat. This QTL has been previously identified across diverse germplasm and agro-ecological zones, including Australia, Canada, China, Japan and Europe (Kato *et al.*, 2001; Li *et al.*, 2004; Mares *et al.*, 2005; Torada *et al.*, 2005, 2008; Ogbonnaya *et al.*, 2007; Chen *et al.*, 2008; Cabral *et al.*, 2014; Albrecht *et al.*, 2015). Given its widespread identification and the magnitude of its effect, *Phs-A1* plays a crucial role in providing resilience to pre-harvest sprouting in wheat.

Despite its consistent identification, only Torada *et al.* (2008) had previously validated the effect of *Phs-A1* in independent isogenic material. In this study, we also validated the effect in UK germplasm using isogenic lines. In both the GI and the artificial sprouting test, we show the QTL to be effective only in lines carrying the Alchemy (resistant) haplotype across the entire *barc170*–*wmc420* QTL interval. However, the level of dormancy of these NILs in the GI test was only intermediate to that of Alchemy, unlike in the artificial sprouting test, where NILs showed similar sprouting resistance as Alchemy. This suggests the presence of additional loci controlling seed dormancy in the Alchemy × Robigus cross, which are independent of the 4AL region.

### *Phs-A1* delays the rate of dormancy loss during seed after-ripening

Understanding the mode and timing of expression of QTL is important to inform effective deployment strategies in breeding programmes and further define the underlying mechanisms. Our physiological characterization shows that *Phs-A1* confers sprouting resistance by delaying the rate of dormancy loss during after-ripening (2–4 weeks after harvest ripeness). After-ripening could be described as a period of dry seed storage during which physiological changes within seeds ultimately lead to the release from dormancy (Gao and Ayele, 2014). Some of these physiological changes include non-enzymatic oxidation of mRNA and protein by Reactive Oxygen Species (ROS) giving rise to changes in protein levels, properties and function upon imbibition (Bykova *et al.*, 2011; Gao *et al.*, 2013a; Gao and Ayele, 2014). Also, changes in the transcript level of genes involved in the biosynthesis or signalling of several hormones, including ABA, indole acetic acid, brassinosteroid, ethylene, cytokinin and salicylic acid have been reported (Liu *et al.*, 2013a; Chitnis *et al.*, 2014). When and how these physiological and transcriptional changes are initiated in dry or imbibed seeds are not well understood. However, the strong effect of *Phs-A1* on after-ripening provides an entry point to further understand the molecular pathway regulating seed after-ripening in wheat and possibly other cereals.

### *Phs-A1* maps 0.3 cM distal to the PM19 genes

Fine-mapping of *Phs-A1* to an initial 0.5 cM interval revealed the presence of at least ten genes in the syntenic *Brachypodium* region with varied biological functions. However due to differential gene loss and duplication events between wheat and *Brachypodium* (Faris *et al.*, 2008; Glover *et al.*, 2015), it is possible that these do not represent the complete gene content in wheat. This is supported by the physical map surrounding the *PM19* genes by Barrero *et al.* (2015), which revealed the presence of additional genes besides the ones defined solely by syntenic relationships. Importantly, the high-resolution fine-mapping conclusively excludes at least six of these genes as being causal for *Phs-A1*, including the *OTU Cysteine Protease, ACC oxidase-1, Ubiquitin Conjugating Enzyme, Myosin J, PM19-A1* and *PM19-A2* genes. These results are particularly surprising for the *PM19* genes, but data from three independent experiments across two different mapping populations showed that *Phs-A1* maps 0.3 cM distal to the *PM19* locus in at least 16 recombinant lines (15 lines in Fig. 5 and one additional line in Supplementary Table S5). Based on these results, we argue that the *PM19* genes are not the main cause of the *Phs-A1* effect, at least in UK wheat varieties. In further support of this conclusion, Torada *et al.* (2008) mapped *Phs-A1* 0.5 cM distal to *xhbe03* (located in the 3ʹ UTR of *PM19-A2*) in two independent populations derived from Japanese and Canadian germplasm. The results of Torada *et al.* (2008) are equivalent to those presented here and suggest that the two *PM19* loci are not the causal genes defining the 4AL sprouting resistance in the UK and possibly other germplasm pools.

The discrepancy, with the results obtained by Barrero *et al.* (2015), could be explained by a number of factors. Firstly, although the *PM19* genes affect seed dormancy in wheat, it is possible that they do not account for the natural variation in sprouting mapped to *Phs-A1* but are instead closely linked to the causal locus. The transgenic data reported by Barrero *et al.* (2015) convincingly support the role of the *PM19* genes in promoting primary seed dormancy in wheat. However, the genetic evidence presented by Barrero *et al.* (2015) is based on the phenotype of only one of five heterogeneous inbred families (F1038) developed from a four-parent MAGIC population. Given the diverse nature of this F_7_ MAGIC population, it is possible that the phenotype of the F1038 family might have been conditioned by other loci that are independent of *Phs-A1*, but were not accounted for in the study. Indeed, there is evidence of this occurring in this population: the phenotype of another heterogeneous inbred family presented in the study (F1516) did not completely support the *PM19* genes as the causal genes.

Alternatively, it is possible that the 4AL PHS resistance originates from two independent natural variants in the closely linked *PM19* and *Phs-A1* loci. This scenario would be reminiscent of the *VRN1* locus controlling vernalization requirement and flowering in wheat (Yan *et al.*, 2003). The *VRN1* locus contains three closely linked genes: *APETALA1 (AP1*), *AGAMOUS – LIKE GENE 1 (AGLG1*) and *Phytochrome C* (*PHYC*), which all function in transitioning to flowering in Arabidopsis (Irish and Sussex, 1990; Flanagan and Ma, 1994; Balasubramanian *et al.*, 2006; Preston *et al.*, 2009; Heijmans *et al.*, 2012). Detailed genetic, expression and sequence analysis initially showed *AP1* to be the main gene underlying *VRN1.* However, recent evidence suggests that *PHYC* is also important in accelerating flowering in wheat under long day conditions (Chen *et al.*, 2014). Analogously, allelic variation at both *PM19* genes and the *Phs-A1* locus could determine resistance to sprouting, but distinct allelic variants could have been selected in Australian and UK germplasm. If true, this would account for the widespread identification of the wider *Phs-A1* locus in diverse germplasms around the world.

In support of this multiple causal gene hypothesis, we identified similar but distinct polymorphisms in the *PM19* genes in our experimental populations compared to those reported by Barrero *et al.* (2015). In the sequence of *PM19-A1* (Supplementary Fig. S4), we found only six of the seven SNPs reported between the dormant (Yitpi) and one of the non-dormant (Chara) parents used by Barrero *et al.* (2015), with only one of these leading to amino acid change between our contrasting parents (Alchemy/Option and Robigus/Clare). We also found a 12-bp deletion close to the translation stop codon in our dormant parent which resulted in the loss of four amino acid residues. This deletion was not reported in the Australian germplasm. Similar haplotype differences were observed in the *PM19-A2* sequence (Supplementary Fig. S5). A smaller deletion polymorphism (188bp) was found in the promoter sequence of *PM19-A2* in UK germplasm compared to the 216bp deletion in Australian lines. Likewise, only one SNP was identified in the coding sequence of *PM19-A2* by Barrero *et al.* (2015), while we identified nine SNPs of which three were non-synonymous. This clearly suggests differences in haplotype structure in these genes between Australian and UK germplasm. This might have functional implications on the regulation and role of *PM19* in these different germplasm pools.

Based on the present study, it is difficult to determine which of these hypotheses is true. We envisage that similar high-resolution fine-mapping of the *Phs-A1* locus in different genetic backgrounds might help resolve this discrepancy. Importantly, the phenotypic characterization of the onset of PHS resistance, as well as the marker resources developed in this study, will facilitate comparative studies across other germplasm, helping clarify the association of the *PM19* gene with the *Phs-A1* natural allelic variation. If the multiple causal gene hypothesis is established, it would also be interesting to test the epistatic interaction between *PM19* and *Phs-A1* by examining lines with recombinant and non-recombinant Yipti and Alchemy/Option haplotypes between the two loci.

### Towards the identification of *Phs-A1* causal gene

In three independent fine-mapping experiments (sprouting experiment-3 to -5), *Phs-A1* showed complete linkage to three genes: *ERF-1B-Like, ASC1* and *PP1-Like*. None of the genes has previously been implicated in the control of dormancy and the induction of germination. *ERF-1B-Like* encodes for an ethylene responsive transcription factor orthologous to Arabidopsis *AtERF1*, which belongs to the AP2/ERF Group-IX family involved in defence response against pathogens (Licausi *et al.*, 2013). *ERF-1B-Like* also shows good homology to *AtERF15*, another member of this AP2/ERF family, which was recently shown to be a positive regulator of ABA response (Lee *et al.*, 2015). ABA is a critical regulator of seed dormancy, with higher seed responsiveness to ABA associated with the PHS resistance. *ASC1* has not been characterized in plants, but sequence analysis of genes containing the ASC-homology domain suggest that they are involved in RNA metabolism as transcription co-activator, RNA processor and regulator of translation (Iyer *et al.*, 2006). *PP1-like* is a member of the serine threonine phosphoprotein phosphatase (PPP), which are involved in a wide range of cellular processes. Since there are likely to be other non-syntenic genes within the interval, it would be premature to suggest these as unique candidate genes for *Phs-A1*. We are currently working to establish a physical map of this interval to reveal other possible candidate genes for *Phs-A1*.

## Supplementary data

Supplementary data are available at *JXB* online.

Fig. S1. PHS resistance QTL on chromosome arm 4AL in the Alchemy × Robigus DH population.

Fig. S2. After-ripening effect of *Phs-A1* in NILs grown at 13 °C post-anthesis.

Fig. S3. Distribution of the sprouting percentage of Option × Claire F_4_ RILs in sprouting experiment-3.

Fig. S4. Alignment of *PM19-A1* coding sequences of UK and Australian germplasm.

Fig. S5. Alignment of *PM19-A2* coding sequences of UK and Australian germplasm.

Table S1. Statistical comparison of the GI and sprouting phenotype of Alchemy × Robigus NILs.

Table S2. Information on genes found in the syntenic *Phs-A1* intervals in wheat and *Brachypodium*.

Table S3. KASP SNP assays used to fine map *Phs-A1*.

Table S4. Statistical comparison of the sprouting scores of Option × Claire RILs in sprouting experiment-3.

Table S5. Statistical comparison of the sprouting score of Option × Claire RILs in sprouting experiment-4.

Table S6. Statistical comparison of the sprouting score of Alchemy × Robigus RILs in sprouting experiment-5.

Supplementary Data
