# Supplementary material for: The wheat Phs-A1 pre-harvest sprouting resistance locus delays the rate of seed dormancy loss and maps 0.3 cM distal to the PM19 genes in UK germplasm
Source: J Exp Bot. 2016 May 23;67(14):4169–78. doi: 10.1093/jxb/erw194 (PMC5301926; doi:10.1093/jxb/erw194)
Supplement: Supplementary Data [file supp_67_14_4169__index.html]

The wheat Phs-A1 pre-harvest sprouting resistance locus delays the rate of seed dormancy loss and maps 0.3 cM distal to the PM19 genes in UK germplasm — The wheat Phs-A1 pre-harvest sprouting resistance locus delays the rate of seed dormancy loss and maps 0.3 cM distal to the PM19 genes in UK germplasm — The wheat Phs-A1 pre-harvest sprouting resistance locus delays the rate of seed dormancy loss and maps 0.3 cM distal to the PM19 genes in UK germplasm — Supplementary Data 

# The wheat *Phs-A1* pre-harvest sprouting resistance locus delays the rate of seed dormancy loss and maps 0.3 cM distal to the *PM19* genes in UK germplasm

## Supplementary Data

Data files

- supplementary\_figures\_S1\_S5\_Tables\_S1\_S6.pdf - Supplementary Data
