# Supplementary material for: The wheat Phs-A1 pre-harvest sprouting resistance locus delays the rate of seed dormancy loss and maps 0.3 cM distal to the PM19 genes in UK germplasm
Source: J Exp Bot. 2016 May 23;67(14):4169–78. doi: 10.1093/jxb/erw194 (PMC5301926; doi:10.1093/jxb/erw194)
Supplement: Supplementary Data [file supp_erw194_supplementary_figures_S1_S5_Tables_S1_S6.pdf]

# Supplementary Data

# The wheat *Phs-A1* pre-harvest sprouting resistance locus delays dormancy loss during seed after-ripening and maps 0.3 cM distal to the *PM19* genes in UK germplasm.

Oluwaseyi Shorinola, Nicholas Bird, James Simmonds, Simon Berry, Tina Henriksson, Peter Jack, Peter Werner, Tanja Gerjets, Duncan Scholefield, Barbara Balcárková, Miroslav Valárik, Michael J Holdsworth, John Flintham, and Cristobal Uauy.

**Fig S1:** PHS resistance QTL on chromosome arm 4AL in the Alchemy x Robigus DH population.

**Fig S2**: After-ripening effect of Phs-A1 in NILs grown at 13 °C post-anthesis.

**Fig S3:** Distribution of the sprouting percentage of Option x Claire F4 RILs in sprouting experiment 3.

**Fig S4**: Alignment of *PM19-A1* coding sequences of UK and Australian germplasm.

**Fig S5:** Alignment of *PM19-A2* coding sequences of UK and Australian germplasm.

**Table S1:** Statistical comparison of the GI and sprouting phenotype of Alchemy x Robigus NILs.

**Table S2:** Information on genes found in the syntenic *Phs-A1* intervals in wheat and *Brachypodium*.

**Table S3:** KASP SNP assays used to fine map *Phs-A1*.

**Table S4:** Statistical comparison of the sprouting scores of Option x Claire RILs in sprouting experiment 3.

**Table S5:** Statistical comparison of the sprouting score of Option x Claire RILs in sprouting experiment 4.

**Table S6:** Statistical comparison of the sprouting score of Alchemy x Robigus RILs in sprouting experiment 5.

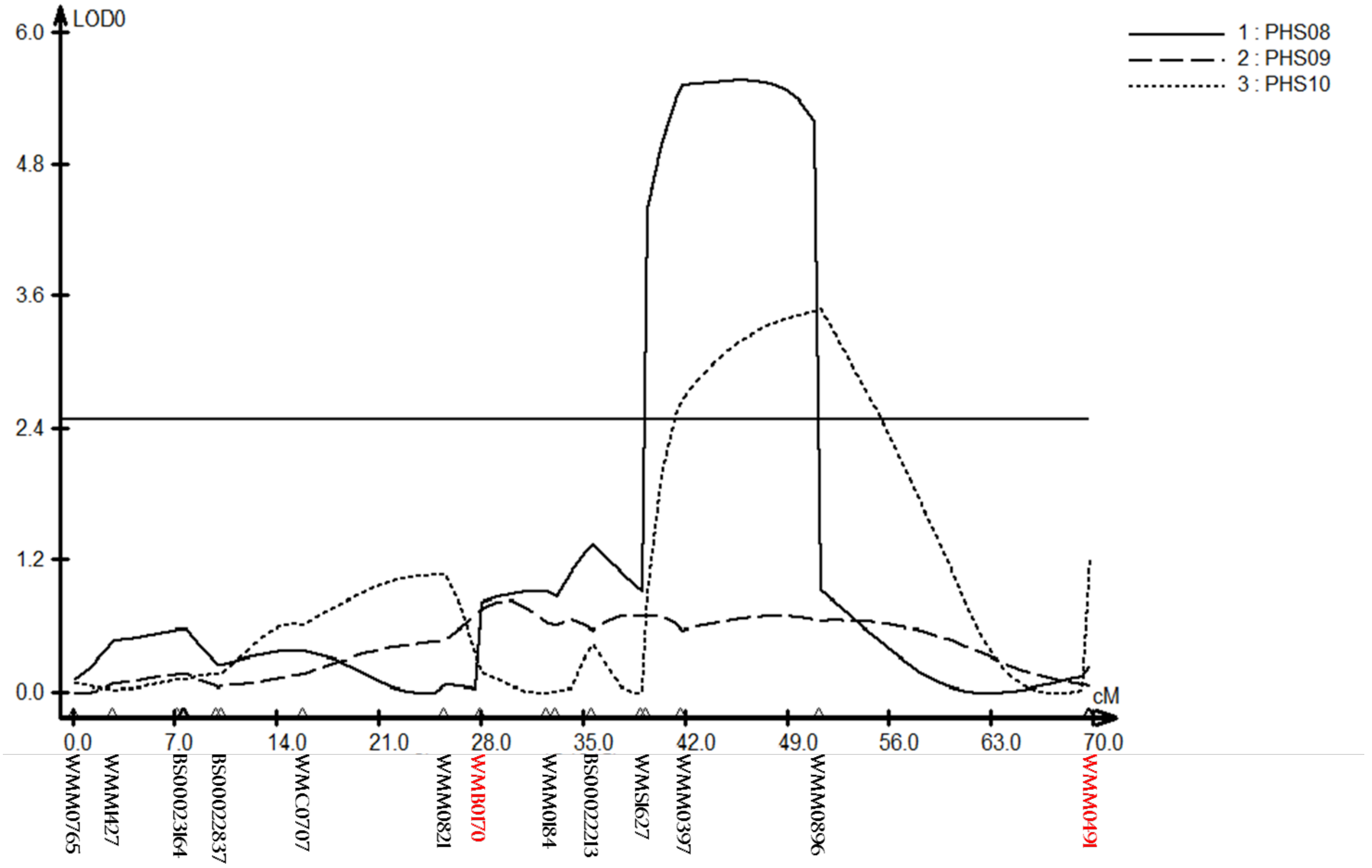


**Fig S1: PHS resistance QTL on chromosome arm 4AL in the Alchemy x Robigus DH population.** A QTL for PHS resistance was identified from composite interval mapping of the arcsin transformed sprouting percentage of DH lines phenotyped across three years of field trial (2008, 2009 and 2010). QTL was detected in 2008 and 2010 but not in 2009. The flanking markers used for selecting the QTL are highlighted in red.

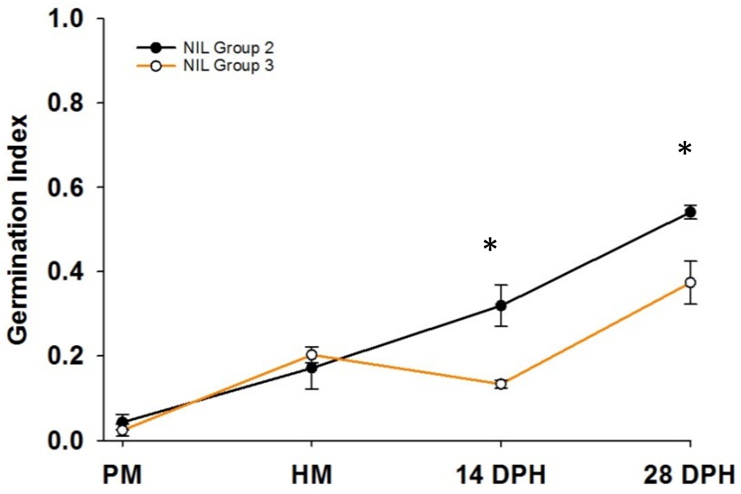


**Fig S2**: **After-ripening effect of *Phs-A1* in NILs grown at 13 °C post-anthesis**. The germination index of seeds harvested from NILs with either the recombinant haplotype (NIL Group 2) or Alchemy-type haplotype (NIL Group 3) between *barc170* and *wmc420* grown at 13 °C post-anthesis. Seeds were tested at Physiological Maturity (PM), Harvest Maturity (HM; 12 days after PM), 14 and 28 Days Post Harvest (DPH). Error bars represent SEM of 3 biological replications for each time point. Significant differences between NILs at P < 0.05 (*) is indicated

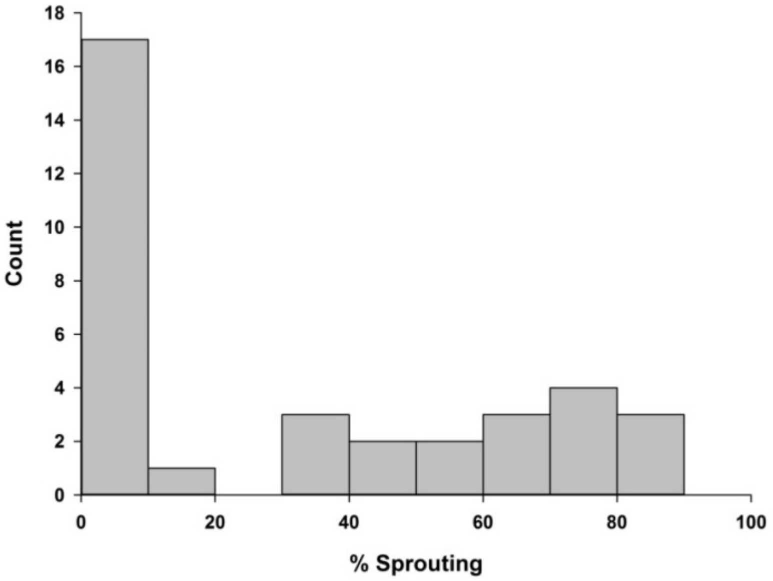


**Fig S3: Distribution of the sprouting percentage of Option x Claire F4 RILs in sprouting experiment 3.**

```
PM19-A1_Alchemy     ATGGCGGGCGTGGGTCGCAACATGGTGGCGCCGCTGCTGGTGCTGAACCTCATCATGTACCTCATCGTCATCGGCTTCGCGAGCTGGAACCTCAACCACTTCATCAACGGCCTCACCAAC 120
PM19-A1_Option      ATGGCGGGCGTGGGTCGCAACATGGTGGCGCCGCTGCTGGTGCTGAACCTCATCATGTACCTCATCGTCATCGGCTTCGCGAGCTGGAACCTCAACCACTTCATCAACGGCCTCACCAAC 120
PM19-A1_Yitpi       ATGGCGGGCGTGGGTCGCAACATGGTGGCGCCGCTGCTGGTGCTGAACCTCATCATGTACCTCATCGTCATCGGCTTCGCGAGCTGGAACCTCAACCACTTCATCAACGGCCTCACCAAC 120
PM19-A1_Claire      ATGGCGGGCGTTGGGCGTAACATGGTGGCGCCGCTGCTGGTGCTGAACCTCATCATGTACATCATCGTCATAGGGTTCGCGAGCTGGAACCTCAACCACTTCATCAACGGCCTCACCAAC 120
PM19-A1_Robigus     ATGGCGGGCGTTGGGCGTAACATGGTGGCGCCGCTGCTGGTGCTGAACCTCATCATGTACATCATCGTCATAGGGTTCGCGAGCTGGAACCTCAACCACTTCATCAACGGCCTCACCAAC 120
PM19-A1_Chara       ATGGCGGGCGTTGGGCGTAACATGGTGGCGCCGCTGCTGGTGCTGAACCTCATCATGTACATCATCGTCATAGGGTTCGCGAGCTGGAACCTCAACCACTTCATCAACGGCCTCACCAAC 120
                    *********** ** ** ******************************************.***********.** ********************************************

PM19-A1_Alchemy     CGCCCCGGCGTCGGCGGCAACGGCGCCACCTTCTACTTCCTCGTCTTCGCCATCCTCGCCGGGGTCGTGGGCGCCGCATCCAAGCTCGCCGGCGTGCACCACGTCCGCACCTGGCGCGGC 240
PM19-A1_Option      CGCCCCGGCGTCGGCGGCAACGGCGCCACCTTCTACTTCCTCGTCTTCGCCATCCTCGCCGGGGTCGTGGGCGCCGCATCCAAGCTCGCCGGCGTGCACCACGTCCGCACCTGGCGCGGC 240
PM19-A1_Yitpi       CGCCCCGGCGTCGGCGGCAACGGCGCCACCTTCTACTTCCTCGTCTTCGCCATCCTCGCCGGGGTCGTGGGCGCCGCATCCAAGCTCGCCGGCGTGCACCACGTCCGCACCTGGCGCGGC 240
PM19-A1_Claire      CGCCCCGGCGTCGGCGGCAACGGCGCCACCTTCTACTTCCTCGTCTTCGCCATCCTCGCCGGGGTCGTGGGCGCCGCATCCAAGCTCGCCGGCGTGCACCACGTCCGCACCTGGCGCGGC 240
PM19-A1_Robigus     CGCCCCGGCGTCGGCGGCAACGGCGCCACCTTCTACTTCCTCGTCTTCGCCATCCTCGCCGGGGTCGTGGGCGCCGCATCCAAGCTCGCCGGCGTGCACCACGTCCGCACCTGGCGCGGC 240
PM19-A1_Chara       CGCCCCGGCGTCGGCGGCAACGGCGCCACCTTCTACTTCCTCGTCTTCGCCATCCTCGCCGGGGTCGTGGGCGCCGCATCCAAGCTCGCCGGCGTGCACCACGTCCGCACCTGGCGCGGC 240
                    ************************************************************************************************************************

PM19-A1_Alchemy     GACAGCCTCGCCACCTCCGCGTCCTCCTCGCTGGTGGCCTGGGCGATCACCGCCTTGGCGTTCGGGCTGGCGTGCAAGGAGATCCACATCGGCGGGTACCGAGGGTGGCGCCTCCGGGTG 360
PM19-A1_Option      GACAGCCTCGCCACCTCCGCGTCCTCCTCGCTGGTGGCCTGGGCGATCACCGCCTTGGCGTTCGGGCTGGCGTGCAAGGAGATCCACATCGGCGGGTACCGAGGGTGGCGCCTCCGGGTG 360
PM19-A1_Yitpi       GACAGCCTCGCCACCTCCGCGTCCTCCTCGCTGGTGGCCTGGGCGATCACCGCCTTGGCGTTCGGGCTGGCGTGCAAGGAGATCCACATCGGCGGGTACCGAGGGTGGCGCCTCCGGGTG 360
PM19-A1_Claire      GACAGCCTCGCCACCTCCGCGTCCTCCTCGCTGGTGGCCTGGGCGATCACCGCCTTGGCGTTCGGGCTGGCGTGCAAGGAGATCCACATCGGCGGGTACCGAGGGTGGCGCCTCCGGGTG 360
PM19-A1_Robigus     GACAGCCTCGCCACCTCCGCGTCCTCCTCGCTGGTGGCCTGGGCGATCACCGCCTTGGCGTTCGGGCTGGCGTGCAAGGAGATCCACATCGGCGGGTACCGAGGGTGGCGCCTCCGGGTG 360
PM19-A1_Chara       GACAGCCTCGCCACCTCCGCGTCCTCCTCGCTGGTGGCCTGGGCGATCACCGCCTTGGCGTTCGGGCTGGCGTGCAAGGGGATCCACATCGGCGGGTACCGAGGGTGGCGCCTCCGGGTG 360
                    *******************************************************************************.****************************************

PM19-A1_Alchemy     CTGGAGGCGTTCGTGATTGTTCTCATGTTCACGCAGCTGCTGTACGTGCTGGCGCTGCACTCGGGCCTCTTCGGGAACCAGTTCGGTGGTAACCATGGTGGTGGGTATCCGGCGGAGCAC 480
PM19-A1_Option      CTGGAGGCGTTCGTGATTGTTCTCATGTTCACGCAGCTGCTGTACGTGCTGGCGCTGCACTCGGGCCTCTTCGGGAACCAGTTCGGTGGTAACCATGGTGGTGGGTATCCGGCGGAGCAC 480
PM19-A1_Yitpi       CTGGAGGCGTTCGTGATTGTTCTCATGTTCACGCAGCTGCTGTACGTGCTGGCGCTGCACTCGGGCCTCTTCGGGAACCAGTTCGGTGGTAACCATGGTGGTGGGTATCCGGCGGAGCAC 480
PM19-A1_Claire      CTGGAGGCGTTCGTGATTGTTCTCATGTTCACGCAGCTGCTGTACGTGCTGGCGCTGCACTCGGGCCTCTTCGGGAACCAGTTCGGTGGTAACCATGGTGGTGGGTATCCGGCGGAGCAC 480
PM19-A1_Robigus     CTGGAGGCGTTCGTGATTGTTCTCATGTTCACGCAGCTGCTGTACGTGCTGGCGCTGCACTCGGGCCTCTTCGGGAACCAGTTCGGTGGTAACCATGGTGGTGGGTATCCGGCGGAGCAC 480
PM19-A1_Chara       CTGGAGGCGTTCGTGATTGTTCTCATGTTCACGCAGCTGCTGTACGTGCTGGCGCTGCACTCGGGCCTCTTCGGGAACCAGTTCGGTGGTAACCATGGTGGTGGGTATCCGGCGGAGCAC 480
                    ************************************************************************************************************************

PM19-A1_Alchemy     GCGTACGGCGCAGGCGTCGGCGACCCGCACAACAAGGGCATGGGCATGGGCACCAG------------GGTCTGA 543
PM19-A1_Option      GCGTACGGCGCAGGCGTCGGCGACCCGCACAACAAGGGCATGGGCATGGGCACCAG------------GGTCTGA 543
PM19-A1_Yitpi       GCGTACGGCGCAGGCGTCGGCGACCCGCACAACAAGGGCATGGGCATGGGCACCAG------------GGTCTGA 543
PM19-A1_Claire      GCGTACGGCGCAGGCGTCGGCGACCCGCACAACAAGGGCATGGGCATGGGCACCGGCGGGGTCGCCAGGGTCTGA 555
PM19-A1_Robigus     GCGTACGGCGCAGGCGTCGGCGACCCGCACAACAAGGGCATGGGCATGGGCACCGGCGGGGTCGCCAGGGTCTGA 555
PM19-A1_Chara       GCGTACGGCGCAGGCGTCGGCGACCCGCACAACAAGGGCATGGGCATGGGCACCAG------------GGTCTGA 543
                    ******************************************************.*            *******
```

**Fig S4: Alignment of *PM19-A1* coding sequences of UK and Australian germplasm.** The coding sequences of *PM19-A1* in Alchemy, Option, Robigus, Claire, Yitpi and Chara *PM19-A1* were aligned with Clustal Omega and the polymorphisms are shown in red.

```
PM19-A2_Claire      ATGGCGGGCGTGGGTCGCAACATGGTGGCGCCGCTGCTGGTGCTGAACCTCATCATGTACCTCATCGTCATCGGCTTCGCGAGCTGGAACCTCAACCACTTCATCAACGGCCTCACCAAC 120
PM19-A2_Robigus     ATGGCGGGCGTGGGTCGCAACATGGTGGCGCCGCTGCTGGTGCTGAACCTCATCATGTACCTCATCGTCATCGGCTTCGCGAGCTGGAACCTCAACCACTTCATCAACGGCCTCACCAAC 120
PM19-A2_Chara       ATGGCGGGCGTGGGTCGCAACATGGTGGCGCCGCTGCTGGTGCTGAACCTCATCATGTACCTCATCGTCATCGGCTTCGCGAGCTGGAACCTCAACCACTTCATCAACGGCCTCACCAAC 120
PM19-A2_Yitpi       ATGGCGGGCGTGGGTCGCAACATGGTGGCGCCGCTGCTGGTGCTGAACCTCATCATGTACCTCATCGTCATCGGCTTCGCGAGCTGGAACCTCAACCACTTCATCAACGGCCTCACCAAC 120
PM19-A2_Alchemy     ATGGCGGGCGTGGGTCGCAACATGGTGGCGCCGCTGCTGGTGCTGAACCTCATCATGTACCTCATCGTCATCGGCTTCGCGAGCTGGAACCTCAACCACTTCATCAACGGCCTCAATAAC 120
PM19-A2_Option      ATGGCGGGCGTGGGTCGCAACATGGTGGCGCCGCTGCTGGTGCTGAACCTCATCATGTACCTCATCGTCATCGGCTTCGCGAGCTGGAACCTCAACCACTTCATCAACGGCCTCACCAAC 120
                    *******************************************************************************************************************. ***

PM19-A2_Claire      CGCCCCGGCGTCGGCGGCAACGGCGCCACCTTCTACTTCCTCGTCTTCGCCATCCTCGCCGGGGTCGTGGGCGCCGCATCCAAGCTCGCCGGCGTGCACCACGTCCGCACCTGGCGCGGC 240
PM19-A2_Robigus     CGCCCCGGCGTCGGCGGCAACGGCGCCACCTTCTACTTCCTCGTCTTCGCCATCCTCGCCGGGGTCGTGGGCGCCGCATCCAAGCTCGCCGGCGTGCACCACGTCCGCACCTGGCGCGGC 240
PM19-A2_Chara       CGCCCCGGCGTCGGCGGCAACGGCGCCACCTTCTACTTCCTCGTCTTCGCCATCCTCGCCGGGGTCGTGGGCGCCGCATCCAAGCTCGCCGGCGTGCACCATGTCCGCACCTGGCATGGG 240
PM19-A2_Yitpi       CGCCCCGGCGTCGGCGGCAACGGCGCCACCTTCTACTTCCTCGTCTTCGCCATCCTCGCCGGGGTCGTGGGCGCCGCATCCAAGCTCGCCGGCGTGCACCATGTCCGCACCTGGCATGGG 240
PM19-A2_Alchemy     CGCCCCGGCGTCGGCGGCAACGGCGCCACCTTCTACTTCCTCGTCTTCGCCATCCTCGCCGGGGTCGTGGGCGCCGCATCCAAGCTCGCCGGCGTGCACCATGTCCGCACCTGGCATGGG 240
PM19-A2_Option      CGCCCCGGCGTCGGCGGCAACGGCGCCACCTTCTACTTCCTCGTCTTCGCCATCCTCGCCGGGGTCGTGGGCGCCGCATCCAAGCTCGCCGGCGTGCACCATGTCCGCACCTGGCATGGG 240
                    ***************************************************************************************************** *************. **

PM19-A2_Claire      GACAGCCTCGCCACCTCCGCGTCCTCCTCGCTGGTGGCCTGGGCGATCACCGCCTTGGCGTTCGGGCTGGCGTGCAAGGAGATCCACATCGGCGGGTACCGAGGGTGGCGCCTCCGGGTG 360
PM19-A2_Robigus     GACAGCCTCGCCACCTCCGCGTCCTCCTCGCTGGTGGCCTGGGCGATCACCGCCTTGGCGTTCGGGCTGGCGTGCAAGGAGATCCACATCGGCGGGTACCGAGGGTGGCGCCTCCGGGTG 360
PM19-A2_Chara       GACAGCCTCGCCACCTCCGCGTCCTCCTCGCTGGTTGCCTGGGCGATTACCGCCTTGGCGTTCGGGCTGGCGTGCAAGGAGATCCACATCGGCGGGTACCGAGGGTGGCGCCTCCGGGTG 360
PM19-A2_Yitpi       GACAGCCTCGCCACCTCCGCGTCCTCCTCGCTGGTTGCCTGGGCGATTACCGCCTTGGCGTTCGGGCTGGCGTGCAAGGAGATCCACATCGGCGGGTACCGAGGGTGGCGCCTCCGGGTG 360
PM19-A2_Alchemy     GACAGCCTCGCCACCTCCGCGTCCTCCTCGCTGGTTGCCTGGGCGATTACCGCCTTGGCGTTCGGGCTGGCGTGCAAGGAGATCCACATCGGCGGGTACCGAGGGTGGCGCCTCCGGGTG 360
PM19-A2_Option      GACAGCCTCGCCACCTCCGCGTCCTCCTCGCTGGTTGCCTGGGCGATTACCGCCTTGGCGTTCGGGCTGGCGTGCAAGGAGATCCACATCGGCGGGTACCGAGGGTGGCGCCTCCGGGTG 360
                    *********************************** *********** ************************************************************************

PM19-A2_Claire      CTGGAGGCGTTCGTCATCATCCTGGCCTTCACGCAGCTGCTCTACGTGCTGGCGCTGCACTCTGGGCTGTTTGGCAACCAGTTTGGTAACCATGCTGGTGGATACGGCGCGGAGCACGGC 480
PM19-A2_Robigus     CTGGAGGCGTTCGTCATCATCCTGGCCTTCACGCAGCTGCTCTACGTGCTGGCGCTGCACTCTGGGCTGTTTGGCAACCAGTTTGGTAACCATGCTGGTGGATACGGCGCGGAGCACGGC 480
PM19-A2_Chara       CTGGAGGCGTTCGTCATCATCCTGGCCTTCACGCAGCTGCTCTACGTGCTGGCGCTGCACTCTGGGCTGTTTGGCAACCAGTTTGGTAACCATGCTGGTGGATACGGCGCGGAGCACGGC 480
PM19-A2_Yitpi       CTGGAGGCGTTCGTCATCATCCTGGTCTTCACGCAGCTGCTCTACGTGCTGGCGCTGCACTCTGGGCTGTTT------------GGTAACCATGCTGGTGGATACGGCGCGGAGCACGGC 468
PM19-A2_Alchemy     CTGGAGGCGTTCGTCATCATCCTGGTCTTCACGCAGCTGCTCTACGTGCTGGCGCTGCACTCTGGGCTGTTT------------GGTAACCATGCTGGTGGATACGGCGCGGAGCACGGC 468
PM19-A2_Option      CTGGAGGCGTTCGTCATCATCCTGGTCTTCACGCAGCTGCT------------------------------------------------------------------------------- 401
                    ************************* ***************

PM19-A2_Claire      GGGTATGGCGCCGGCGATCCGCACAATAAGGGCATGGGCACCGGCGGCGTCGCCAGGGTCTGA 543
PM19-A2_Robigus     GGGTATGGCGCCGGCGATCCGCACAATAAGGGCATGGGCACCGGCGGCGTCGCCAGGGTCTGA 543
PM19-A2_Chara       GGGTATGGCGCCGGCGATCCGCACAATAAGGGCATGGGCACCGGCGGCGTCGCCAGGGTCTGA 543
PM19-A2_Yitpi       GGGTATGGCGCCGGCGATCCGCACAATAAGGGCATGGGCACCGGCGGCGTCGCCAGGGTCTGA 531
PM19-A2_Alchemy     GGGTATGGCGCCGGCGATCCGCACAATAAGGGCATGGGCACCGGCGGCGTCGCCAGGGTCTGA 531
PM19-A2_Option      --------------------------------------------------------------- 401
```

**Fig S5: Alignment of *PM19-A2* coding sequences of UK and Australian germplasm.** The coding sequences of *PM19-A2* in Alchemy, Option, Robigus, Claire, Yitpi and Chara were aligned with Clustal omega and the polymorphisms are shown in red. Partial *PM19-A2* sequence was obtained for Option.

**Table S1: Statistical comparison of GI and sprouting phenotype of Alchemy x Robigus NILs**

| Test | Lines | Mean | Std Error | Sig (P) against Alchemy | Sig (P) against Robigus | Phenotype Classification |
|---|---|---|---|---|---|---|
| GI | Alchemy | 0.1 | 0.01 | NA | < 0.001 | Low |
| | Robigus | 0.9 | 0.02 | < 0.001 | NA | High |
| | Group 1 | 0.8 | 0.06 | < 0.001 | 0.895 | High |
| | Group 2 | 0.9 | 0.01 | < 0.001 | 0.998 | High |
| | Group 3 | 0.4 | 0.05 | < 0.001 | < 0.001 | Moderate |
| | Group 4 | 0.9 | 0.01 | < 0.001 | 0.994 | High |
| | Group 5 | 0.8 | 0.05 | < 0.001 | 0.908 | High |
| | Group 6 | 0.7 | 0.04 | < 0.001 | 0.009 | Moderate |
| | Group 7 | 0.9 | 0.02 | < 0.001 | 0.895 | High |
| % Sprouting Arcsin | Alchemy | 9.0 | 1.68 | - | 0.002 | Low |
| | Robigus | 27.9 | 2.21 | 0.002 | - | High |
| | Group 1 | 39.7 | 3.52 | < 0.001 | 0.044 | High |
| | Group 2 | 27.0 | 2.41 | < 0.001 | 1 | High |
| | Group 3 | 7.5 | 0.98 | 1 | < 0.001 | Low |
| | Group 4 | 49.8 | 3.14 | < 0.001 | < 0.001 | High |
| | Group 5 | 27.5 | 2.27 | < 0.001 | 1 | High |
| | Group 6 | 29.1 | 3.30 | < 0.001 | 1 | High |
| | Group 7 | 31.5 | 2.18 | < 0.001 | 0.92 | High |

**Table S2: Information on genes found in the syntenic *Phs-A1* intervals in wheat and *Brachypodium*.**

| | ***Brachypodium* genes** | **IWGSC CSS Contigs** | **Gene model ID** | **Gene Names** |
|---|---|---|---|---|
| **1** | *Bradi1g00600* | 4AL_7123764 | Traes_4AL_BFAB568BF | *Plasma Membrane Protein 19-A2 (PM19-A1)* |
| | | 4AL_7174272 | Traes_4AL_F99FCB25F | *Plasma Membrane Protein 19-A2 (PM19-A2)* |
| **2** | *Bradi1g00607* | 4AL_7174272<br>4AL_7093716 | Traes_4AL_DD1B27086* | *Myosin-J heavy chain protein* |
| **3** | *Bradi1g00620* | 4AL_ 7074626 | M28059[#] | *Ubiquitin Conjugating Enzyme (UBC)* |
| **4** | Non-collinear | 4AL_ 7074626 | Traes_4AL_65DF744B71 | *1 Aminocyclopropane carboxylate Oxidase-1-like (ACC – Oxidase-1)* |
| **5** | *Bradi1g00670* | 4AL_7095843 | Traes_4AL_F18648C49 | *Ethylene Responsive Factor 1B (ERF-1B)* |
| **6** | *Bradi1g00677* | 4AL_6230861<br>4AL_5621077 | Traes_4AL_02AE47773* | *Activating Signal Co-integrator 1 Like (ASC1-Like)* |
| **7** | *Bradi1g00690* | 4AL_7159962 | Traes_4AL_2EBB63DBA | *Serine threonine Protein Phosphatase 1-like (PP1-Like)* |
| **8** | *Bradi1g00700* | 4AL_7162562 | Traes_4AL_C56125840 | *Phosphate Transporter* |
| **9** | *Bradi1g00710* | 4AL_7177172 | Traes_4AL_DD344FC87 | *Hypothetical protein* |
| **10** | *Bradi1g00720* | 4AL_7172877 | Traes_4AL_F00707FAF | *OTU cysteine Protease* |

*Gene model is split across two contigs.
[#] NCBI gene model used as no suitable gene model was found on EnsemblPlant

**Table S3: KASP SNP assays used to fine map *Phs-A1***

| Gene | Robigus/Claire Specific Primer | Alchemy/Option Specific Primer | Common Primer |
|---|---|---|---|
| *PM19_A2* | GCACTCTGGGCTGTTTGGc | GCACTCTGGGCTGTTTGGt | TATCAGCCTGTCACTGTCGC |
| *ACC Oxidase-1* | CTCGTTGAAGCGCCGGAt | CTCGTTGAAGCGCCGGAc | CCAGGTGACGAACCACGA |
| *PP1-like* | GTCCTAGAGTTGAGACACTAATTTTAa | GTCCTAGAGTTGAGACACTAATTTTAg | AGACTTAACCAAGTCTCAGTCA |
| *OTU Cysteine Protease* | ACCGTTTCGGTTTCTTGTTc | ACCGTTTCGGTTTCTTGTTg | TTTGAAGAAAAATGTTCGCAGA |
| *STW17* | TTGCAGTCATGGTGTGATAAc | TTGCAGTCATGGTGTGATAAt | GCTAGCCACACACCGTCT |

**Table S4: Statistical comparison of the sprouting scores of Option x Claire RILs in sprouting experiment 3.**

| Groups | line | % Sprouting | Std Error | Sig (P) against Option | Sig (P) against Claire | Phenotype Classification |
|---|---|---|---|---|---|---|
| Susceptible Parent | Claire | 70.6 | 8.3 | < 0.001 | NA | High |
| Resistant Parent | Option | 2.4 | 2.0 | NA | < 0.001 | Low |
| Claire Control RIL | - | 65.0 | 3.7 | < 0.001 | 1.000 | High |
| Option Control RIL | - | 4.0 | 0.6 | 0.992 | < 0.001 | Low |
| RIL Group 11 | 4/204 | 0.0 | 0.0 | 1.000 | < 0.001 | Low |
| RIL Group 12 | 20/24 | 60.0 | 4.4 | < 0.001 | 0.800 | High |
| | 24/28 | 75.0 | 7.9 | < 0.001 | 1.000 | High |
| | 25/58 | 36.2 | 4.1 | < 0.001 | 0.004 | Moderate |
| | 4/95 | 74.5 | 5.3 | < 0.001 | 1.000 | High |
| RIL Group 13 | 21/46 | 4.6 | 1.0 | 0.856 | < 0.001 | Low |
| RIL Group 14 | 20/42 | 49.4 | 8.4 | < 0.001 | 0.235 | High |
| | 21/19 | 34.2 | 3.9 | < 0.001 | 0.002 | Moderate |
| | 22/57 | 73.5 | 4.3 | < 0.001 | 1.000 | High |
| | 26/8 | 37.2 | 7.2 | < 0.001 | 0.005 | Moderate |
| RIL Group 15 | 5/189 | 88.1 | 3.3 | < 0.001 | 0.640 | High |

**Table S5: Statistical comparison of the sprouting score of Option x Claire RILs in sprouting experiment 4.**

| Groups | Line | % Sprouting | Std Error | Sig (P) against Option | Sig (P) against Claire | Phenotype Classification |
|---|---|---|---|---|---|---|
| Susceptible Parent | Claire | 89.9 | 2.6 | < 0.001 | NA | High |
| Resistant Parent | Option | 0.7 | 0.2 | NA | < 0.001 | Low |
| RIL Group 11 | 27/8 | 32.1 | 4.6 | < 0.001 | < 0.001 | Moderate |
| | 4/204 | 50.7 | 7.6 | < 0.001 | 0.001 | Moderate |
| RIL Group 12 | 20/24 | 93.6 | 1.0 | < 0.001 | 1 | High |
| | 24/28 | 95.4 | 0.8 | < 0.001 | 0.982 | High |
| | 25/58 | 97.8 | 0.2 | < 0.001 | 0.917 | High |
| | 4/95 | 96.4 | 1.2 | < 0.001 | 0.884 | High |
| RIL Group 13 | 21/46 | 49.8 | 7.3 | < 0.001 | 0.024 | Moderate |
| | 26/37 | 69.6 | 5.7 | < 0.001 | 0.472 | High |
| RIL Group 14 | 20/42 | 97.4 | 0.5 | < 0.001 | 0.596 | High |
| | 21/19 | 72.0 | 6.8 | < 0.001 | 0.375 | High |
| | 22/57 | 95.0 | 0.9 | < 0.001 | 0.969 | High |
| | 26/8 | 91.1 | 1.6 | < 0.001 | 1 | High |

**Table S6: Statistical comparison of the sprouting score of Alchemy x Robigus RILs in sprouting experiment 5.**

| Groups | Line | % Sprouting | Std Error | Sig (P) against Option | Sig (P) against Claire | Phenotype Classification |
|---|---|---|---|---|---|---|
| Susceptible Parent | Robigus | 84.4 | 3.3 | 0 | NA | High |
| Resistant Parent | Alchemy | 18.5 | 7.8 | NA | 0 | Low |
| RIL Group 21 | 3/70/3/27 | 15.9 | 0.8 | 0.982 | 0.005 | Low |
| | 3/101/6/31 | 9.0 | 0.9 | 1 | 0.001 | Low |
| RIL Group 22 | 3/63/1/22 | 86.4 | 0.9 | 0 | 1 | High |
| | 3/70/2/94 | 77.5 | 5.6 | 0.001 | 1 | High |
| | 3/72/3/54 | 76.6 | 5.8 | 0.001 | 1 | High |
| | 3/72/4/41 | 88.1 | 4.1 | 0 | 1 | High |
| | 3/101/9/23 | 78.4 | 5.8 | 0.001 | 1 | High |
| | 3/101/9/73 | 90.5 | 3.1 | 0 | 1 | High |
| | 3/124/6/81 | 84.2 | 6.0 | 0 | 1 | High |
| | 3/158/1/82 | 92.2 | 2.2 | 0 | 0.992 | High |
| | 3/158/2/67 | 93.3 | 1.7 | 0 | 0.984 | High |
| | 3/175/1/60 | 89.7 | 1.1 | 0 | 1 | High |
| RIL Group 23 | 3/101/6/48 | 2.6 | 0.9 | 0.92 | 0 | Low |
| RIL Group 24 | 3/148/2/4 | 5.4 | 0.3 | 1 | 0 | Low |
| | 3/172/10/92 | 11.4 | 7.5 | 0.986 | 0 | Low |
| RIL Group 25 | 3/106/9/2 | 81.7 | 4.2 | 0 | 1 | High |
| | 3/124/6/24 | 80.6 | 7.7 | 0.001 | 1 | High |
| | 3/124/6/74 | 93.9 | 1.6 | 0 | 0.617 | High |
| | 3/149/9/55 | 90.1 | 3.9 | 0 | 0.87 | High |
| | 3/158/1/38 | 88.5 | 5.7 | 0 | 1 | High |
| | 3/175/1/50 | 88.9 | 2.3 | 0 | 1 | High |
